# Modern Contraceptive Utilization and Associated Factors among Married Gumuz Women in Metekel Zone North West Ethiopia

**DOI:** 10.1155/2020/8010327

**Published:** 2020-07-23

**Authors:** Alemnew Ashagrie Adane, Yibeltal Alemu Bekele, Ergoye Melese, Getasew Taddesse Worku, Henok Biresaw Netsere

**Affiliations:** ^1^Department of Epidemiology and Biostatistic, Pawi Health Science College, Ethiopia; ^2^Department of Reproductive Health and Population Studies, School of Public Health, Bahir Dar University, Bahir Dar, Ethiopia; ^3^Department of Epidemiology and Biostatistic, School of Public Health, Bahir Dar University, Bahir Dar, Ethiopia; ^4^Department of Health Service Management and Health Economics, School of Public Health, Bahir Dar University, Bahir Dar, Ethiopia; ^5^Department of Nursing, School of Health science, Bahir Dar University, Bahir Dar, Ethiopia

## Abstract

*Background*. Modern contraceptives are a key intervention to improve the health of both the mother and children by preventing unintended pregnancy. However, significant numbers of women were facing abortion-related morbidity and mortality globally including Ethiopia due to the nonuse or failure of contraceptive uses. Therefore, the aim of this study was to assess the utilization of modern contraceptive methods and associated factors among married Gumuz women in Metekel Zone North West Ethiopia. A community-based cross-sectional study was conducted among 580 women from March 1 to 30/2019. Pretested structured interview administer questionnaires was used to collect the data. Data were cleaned, coded, and entered into Epi-info version 7.1 and export to SPSS for farther analysis. Both bivariate and multivariate analyses were used. On bivariate analysis *P* value, less than 0.2 were used to select the candidate variable for multivariate analysis. *P* value and 95% confidence interval were used to measure the level of significance on multivariate analysis and those variables whose *P* value < 0.05 were considered as statically significant. The prevalence of modern contraceptive method was 18.6% [95% CI: 15.00-22.00]. Age ≥ 35 year AOR 4.67; 95% CI (1.34 -16.18), able to read and write AOR 6.45 95% CI(2.98-13.97), primary school AOR 6.56; 95% CI (2.22-19.38), secondary school AOR 7.27; 95% CI (3.00 -17.61), counseled on contraceptive methods AOR 3.72 95% CI (2.11-6.56), moderate knowledge on modern contraceptive method AOR 2.31; 95% CI (1.15-4.64), and good knowledge on modern contraceptive method AOR 4.37; 95% CI (2.38-8.02) were identified as statistically significant with modern contraceptive methods utilization. The prevalence of contraceptive utilization was low when compared to the national and the regional figure. Maternal age, maternal educational status, counseling about modern contraceptive methods, and knowledge on modern contraceptive methods were found as statistically significant with modern contraceptive utilization.

## 1. Background

Family planning (FP) is defined as the ability of individuals or couples to anticipate and attain their desired number of children, the spacing, and timing of their births [1]. In this concern, the modern contraceptive method key decides couples to have sexual intercourse at any time [2]. Nowadays, more attention is being given for family planning because of their multiple benefits to improve both the health of children and woman's by reducing the risk of unintended pregnancies. Family planning is one of the main interventions to reduce maternal and neonatal morbidity and mortality [3, 4].

World Health Organization (WHO) report indicates that using modern contraceptive can reduce 32% of all maternal death and nearly 10% of childhood deaths [5]. It is also estimated that 90% of the abortion-related and 20% of the pregnancy-related morbidity and mortality can be prevented with the use of effective family planning methods [6]. However, 225 million women who want to prevent or delay pregnancy but face significant barriers to obtaining and using modern contraceptive methods that meet their individual needs [7].

Globally, the trends of modern contraceptive utilization have increased slightly, which was 54% in 1990 to 57.4% in 2015. While in Africa, the trends of modern contraceptive utilization have ascended a little, which increases from 23.6% in 2008 to 28.5% in 2015 [8, 9].

Studies conducted in Asia show that the magnitudes of modern contraceptive utilization among married women were 36%, 81.27%, in Nepal and Bangladesh, respectively [10, 11]. Similarly, studies in Africa show that the magnitudes of modern contraceptive utilization were 39.9%, 32%, 31%, and 92.7% in South Africa, Baringo Kenya, and Nigeria, respectively [12–14]. In addition, different literature conducted in Ethiopia also shows that the prevalence of modern contraceptive utilization ranges from 9.1%-46.9% [15–22].

Studies conducted in developing countries showed that the age of the respondent, education status of the respondent, parity, religion, marital status, knowledge about modern family planning method and side effects, method approval by self and partners, and employment status were factors for the utilization of contraceptives [23–25]. Similarly, studies in Ethiopia shows age, residence, educational status of the mother, couple discussion, perceived husband approval, discussion with health extension workers, perceived cultural acceptance, the desire of more children, monthly income, and numbers of living children were determined by modern family planning method utilization [15, 17, 19, 20].

According to the Ethiopian mini demographic health survey, the 2019 report indicates that the contraceptive prevalence rate was 41% while the 2015/16 Ethiopian health sector transformation plan was planned to achieve contraceptive prevalence rate to 55% in 2019/20 [26, 27]. There is some increment on contraceptive prevalence rate while the increment was not sufficient enough to achieve the country planned in 2019/20 on the area of health sector transformation plan.

There are few studies about prevalence and associated factors of modern contraceptive utilization among married Gumuz women. But these studies were fragmented and not context-specific in the study area because Gumuz ethnic is one of the pastorals groups in Ethiopia. Therefore, this study was conducted to determine the magnitudes and associated factors of modern contraceptive utilization among married Gumuz women.

## 2. Methods and Materials

### 2.1. Study Area

Metekel zone is one of the three zones found in Benishangul Gumuz Regional State, located 570 km Northwest of Addis Ababa. Administratively, the zone is structured into seven districts that are (Pawie, Mandura, Wombera, Debati, Bullen, Dangur, and Guba), having 7 urban and 97 rural villages. Based on the 2007 E.C Ethiopian census, the total population of the zone was estimated to be 276,367, of whom 139,119 were men and 137,248 women. The majority of 238,752 of the population live in rural areas and economically dependent on farming. Gumuz is the majority ethnic group in the zone followed by Shinasha. The zone has seven hospitals, 13 health centers, and 76 health posts.

### 2.2. Sample Size Determination and Procedures

The sample size was calculated through a single population proportion formula. The proportion of women utilization of family planning services this study used was 64.6% [28], with the consideration of a margin of error of 5%, nonresponse rate of 10%, and design effect of 1.5. The final total sample size was 580 women. A multistage sampling method was used. A total of nine Gumuz villages was included in the zone selected randomly from the seven districts. A preliminary survey was conducted to identify married women in the zone. Finally, a simple random sampling technique was used to identify each participant in each selected village. The number of respondents in each village was assigned proportionally.

### 2.3. Variables of the Study

Dependent Variable: Modern contraceptive utilization means utilizations of any modern contraceptive methods during the data collection period (Yes/No).

Independent Variables were categorized into four themes. Sociodemographic variables: Age, education, religion, and occupation. Reproductive health-related variables: Gravidity, parity, birth interval, desired number of children, and family size. Individual-related variables: Knowledge of FP, attitude towards FP, and couples' decision about FP. Health facility-related variables: Choice of FP method, counseling on contraceptive method, and distance of health facility.

### 2.4. Operational Definition

Knowledge about modern family planning method: Based on bloom cutoff point those reproductive-age women who answered 75-100% 50-74%, and <50% from seven knowledge assessing questions were considered as having good knowledge, moderate knowledge, and poor knowledge, respectively.

Attitude towards modern contraceptive methods: Based on the bloom cutoff point, those reproductive-age women who answered 75-100%, 50-75%, and <50% from eight attitude assessing questions were considered as having a positive attitude, neutral attitude, and negative attitude, respectively.

### 2.5. Data Collection Tools and Procedures

The questionnaire was developed by reviewing different literature. It was first prepared in English and translated into Amharic then translated back to English to check for consistency. Data were collected via interview. Trained health extension workers one in each village with three supervisors those were nurses were assigned for data collection along with the principal investigator.

### 2.6. Data Quality Controls

The questionnaire was pretest with 10% (58) of the sample population incomparable district. Amharic versions of the questioner were used to collect the data. Two-day training was provided for the data collectors and supervisors on the objectives and purposes of the study. The supervisors have supervised the data collection process every day and the principal investigator also cheeks the collected questionnaire for completeness every other day.

### 2.7. Data Processing and Analysis

Data were cleaned, coded, and entered into Epi-info version 7.1 then exported to SPSS version 23 for further analysis. A descriptive analysis was carried out to see the distribution of independent variables. Binary logistic regression was used to examine associations between the dependent variable and independent variables. In the bivariate analysis, those variables whose *P* value less than 0.25 was used to select the candidate variable for multivariate analysis. Multicollenearity test was performed via a variance inflation factor before fitted to the final model. *P*values < 0.05 with 95% confidence interval was used to determine the level of significance on multivariable analysis. Hosmer and Lemeshow test were used for checking the model fitness for logistic regressions at *P* value > 0.05.

## 3. Results

### 3.1. Sociodemographic Characteristics

A total of 580 married women participated. Four hundred three (69.5%) of the participants were in the age group of 25 to 34 years with the mean age of 32.79 (SD±5.53). Four hundred eighty-three (83.5%) of the respondents were not able to read and write, while only 54 (9.3%) of the respondents attained formal education. Five hundred eleven (88.1%) respondents were farmers. Two hundred twenty-eight (39.4%) respondents were followers of pagans (Table 1).

### 3.2. Reproductive History

Three hundred twenty-four (55.9%) respondents were married before the age of 15 years. Two hundred seventeen-nine (48.1%) respondents had planned to have an additional child within 2 years periods. One hundred twenty-nine (22.2%) respondents had discussed on contraceptive methods with their partners. Four hundred seventeen (71.9%) respondents said that only partners decided the numbers of their children (Table 2).

### 3.3. Individual Related Factors

Two hundred eight (46.2%) respondents had heard about family planning methods. Three hundred eighty (65.5%) respondents had had poor knowledge, 99 (15.4%) respondents had had moderate knowledge, and 111(19.1%) respondents had had good knowledge about contraceptives methods. The most commonly mentioned methods by respondents were injectable 158 (27.1%), oral contraceptive pills 119 (20.4%), and condom 103 (17.7%) (Table 3).

### 3.4. Prevalence of Current Utilization of Modern Contraceptive Methods

The overall utilizations of modern contraceptive utilization were 108 (18.6%). Among currently using modern contraceptive method seventy-three (12.6%), 20 (3.4%), and 15(2.6%) respondents were using the injectable method, oral contraceptive pills, and implants, respectively. One hundred five (18.1%) respondents who were got a choice modern contraceptive method.

### 3.5. Factors That Associated with Current Utilization of Modern Contraceptive Methods

In the bivariate analysis, maternal education, age, occupation, parity, number of living children, couple discussion, knowledge of FP, and counseling on contraceptive method were candidate variable for multivariate analysis at *P* value 0.25. And on multivariate analysis, maternal education, ages, counseling on contraceptive methods, and knowledge about FP were factors associated with modern contraceptive methods at the *P* value less than 0.05.

The odds of modern contraceptive utilization among women who attained primary school and secondary education were 7 times more likely to utilize modern contraceptive methods than those cannot read and write (AOR = 6.56; 95% CI: 2.22-19.38) and (AOR = 7.27; 95% CI: 3.00-17.61), respectively. The odds of modern contraceptive utilization among women whose age was ≥35 was 5 times more likely to utilize modern contraceptive methods than those women whose ages 15-24 (AOR = 4.67;95% CI: 1.34-16.18). Women who had been counseled on modern contraceptive methods were 4 times higher than those women who did not counsel on modern contraceptives (AOR = 3.72; 95% CI: 2.11-6.56**)**. Women who had good knowledge on modern contraceptive methods were 4 times higher than those women who had poor knowledge (AOR = 4.37; 95% CI: 2.38-8.02) (Table 4).

## 4. Discussion

The prevalence of modern contraceptive utilization was 18.6% with 95% CI [15.00-22.00]. This finding of this study was in line with the studies conducted in the Bale eco region Ethiopia 20.8% (11). However, the finding of this study was lower than studies conducted in Ethiopia and Benishagule Gumuz coverage of FP (35%) and (28%), respectively [27], and Kenya 51% [28]. The discrepancy might be due to sociodemographic differences between the study areas. In this study, only 9.3% of respondents attained formal education; it is lower than the national level, around 59.5% attained formal education [29]. Similarly, 93% of women attained formal education in Kenya [30]. In addition, access to media may increase the utilizations of modern family planning method utilization, while in this study, only 1.2% of women has access to media [31].

The findings of this study show that mothers who had had knowledge about modern family planning were more likely to use family planning services than those who had poor knowledge. This finding was consistent with studies conducted in Tigray, Dabat, and in Farta district [20–27, 32, 33]. This might be due to the knowledge about the modern family planning method is the key means of understanding the prone and cons of each method and decrease the complaints. In addition, knowledge on family planning method decreases the myth related to modern family planning methods and increases the uptakes of modern family planning methods [34].

Mothers who had been counseled on modern contraceptive methods in the health facility were more likely to use modern contraceptive than mothers who had never been counseled. This finding was in line with the findings of previous studies conducted in Benchi-Maji zone, Bale eco region and Mexico [16, 35]. This might be due to counseling helps the women to make informed decisions about family planning services utilization [36]. This implies that strengthening counseling service in the health facility increase the uptakes of modern family planning utilization.

Maternal age was found significantly associated for modern contraceptive utilization specifically from age ≥ 35 were more likely to use contraceptive than those women whose age 15-24 years. This finding was in line with that of Nepal [24]. This might be due to the fact that women in lower groups (15-24 years) might perceive a low risk of pregnancy. In addition, in this study, the odds of modern contraceptive utilization among women who have primary school level education were 7 times and secondary school level education was 7 times higher than those women who cannot read and write. This might be due to education is one of a key factor for women's empowerment and decide to utilize family planning services compare to noneducated women.

## 5. Conclusion

The prevalence of modern contraceptive utilization in the study area was low compared to the national prevalence of the country. Maternal education, maternal ages (≥ 35), knowledge about FP, and counseling on modern contraceptive methods were affecting modern family planning services utilization. Recommendation, for ministry of education, work hard to reach women's educational status at least the primary level.

## Figures and Tables

**Table 1:** Sociodemographic characteristics the respondents in Metekel zone, Benishangul, Ethiopia, 2019 (*n* = 580).

Variables	Category	Frequency	Percent (%)
Age	15-24	34	5.9
	25-34	403	69.5
	≥35	143	24.6

Religion	Orthodox	87	15
	Muslim	64	11
	Protestant	92	16
	Catholic	108	18.6
	Pagan	228	39.4

Occupation	Farmer	511	88.1
	Government employee	43	7.5
	House wife	20	3.4
	Student	6	1

Educational level	Cannot read and write	483	83.3
	Able to read and write	43	7.4
	Primary school (1–8)	19	3.3
	Secondary school (9–12)	35	6

**Table 2:** Reproductive health characteristics of the respondents in Metekel zone, Benishangul, Ethiopia, 2019.

Variable	Category	Frequency	Percent (%)
Age at first marriage	<15	324	55.9
	15-18	254	43.8
	>18	2	0.3

Gravidity	Prime gravid	15	2.6
	Multigravid	371	64
	Grand multi-Para	194	33.4

Parity	Prime Para	11	1.9
	Multi-Para	364	62.8
	Grand multi-Para	205	35.3

Age at first birth	15-19	557	96.0
	20-24	23	4.0

Plan additional children within 2 years	Yes	279	48.1
	No	301	51.9

How many alive children	1	10	1.7
	2-4	369	63.9
	≥5	201	34.7

Discussed with partners on contraceptive method	Yes	129	22.2
	No	451	77.8

Decides number of children	Husband	417	71.9
	Wife	83	14.3
	Both	80	13.8

**Table 3:** Knowledge of the respondent on modern contraceptive methods in Metekel zone, Benishangul, Ethiopia, 2019.

Variables	Category	Frequency	Percent (%)
Have you ever heard about modern contraceptives method?	Yes	268	46.2
	No	312	53.8

Types of modern family planning method you know	Pills	119	20.4
	Depo-Provera	158	27.1
	Implant	73	12.5
	IUCD	10	1.7
	Condom	103	17.7

From whom do you get information on modern contraceptive methods	Health workers	319	55
	Husband	17	2.9
	Neighbors	182	31.4
	School	43	7.4
	Radio	7	1.2

What sources of family planning do you know?	Government HFs	232	40
	Private clinic	76	13.1
	Pharmacy	14	2.4

Do you know modern contraceptives used to unwanted pregnancy?	Yes	257	44.3
	No	323	55.7

Do you know modern contraceptives used to spacy children?	Yes	237	40.9
	No	343	59.1

Do you know early initiation of modern contraceptives after delivery is important	Yes	224	38.6
	No	356	61.4

Counseling about modern family planning method	Yes No	108 472	18.62 81.38

**Table 4:** Factors associated with modern contraceptive methods utilization among married Gumuz women in Metekel zone, Benishangul Gumuz Ethiopia, 2019.

Variables	Modern contraceptive utilization		COR (95% CI)	AOR (95% CI)
	Yes	No		
Age in year				
15-24	8 (7.4)	26 (5.5)	1	1
25-34	64 (59.3)	339 (71.8)	0.61 (0.26-1.41)	1.50 (0.49-4.61)
≥35	36 (33.3)	107 (22.7)	1.09 (0.45-2.63)	4.67 (1.34-16.18)
Educational status				
Cannot read and write	60 (55.6)	423 (89.6)	1	1
Able to read and write	20 (18.5)	23 (4.9)	6.13 (3.17-11.83)	6.4 (2.9-13.9)
Primary school (1–8)	12 (11.1)	7 (1.5)	12.08 (4.57-31.9)	6.56 (2.22-19.38)
Secondary school (9–12)	16 (14.8)	19 (4)	5.93 (2.89-12.17)	7.27 (3.00-17.61)
Parity				
≤1	4 (3.7)	11 (2.3)	1	1
2-4	65 (60.2)	306 (64.8)	0.58 (0.18-1.89)	1.92 (0.92-3.98)
≥5	39 (36.1)	155 (32.8)	0.54 (0.69-0.20)	3.9 (0.51-29.46)
Counseling on contraceptive				
No	78 (16.5)	394 (83.5)	1	1
Yes	57 (52.8)	51 (47.2)	5.64 (3.60-8.84)	3.72 (2.11-6.56)
Knowledge				
Poor knowledge	34 (31.5)	346 (73.3)	1	1
Moderate knowledge	31 (28.7)	58 (12.3)	5.43 (3.10-9.52)	2.31 (1.15-4.46)
Good knowledge	43 (39.8)	68 (14.4)	6.43 (3.82-10.81)	4.37 (2.38-8.02)
Couple discussion				
No	78 (72.2)	373 (79)	1	1
Yes	30 (27.8)	99 (21)	1.44 (.90-2.33)	1.95 (0.51-2.75)
Live children				
≤1	2 (1.9)	8 (1.8)	1	1
2-4	70 (64.8)	299 (63.3)	0.93 (0.19-4.50)	1.46 (0.05-3.32)
≥5	36 (33.3)	165 (35)	0.87 (0.17-4.28)	1.44 (0.05-3.31)

## Data Availability

The data used to support the findings of this study are included within the article.
